# Supply/demand ratio for medical consultations, diagnostic tests and chronic kidney disease monitoring in the Brazilian National Health System: a descriptive study, state of São Paulo, Brazil, 2019

**DOI:** 10.1590/S2237-96222022000200014

**Published:** 2022-07-08

**Authors:** Farid Samaan, Marcelo Gutierrez, Gianna Mastroianni Kirsztajn, Ricardo Cintra Sesso

**Affiliations:** 1 Secretaria de Estado da Saúde de São Paulo, Grupo de Planejamento e Avaliação, São Paulo, SP, Brazil; 2 Universidade Federal de São Paulo, Faculdade de Medicina, São Paulo, SP, Brazil

**Keywords:** Renal Insufficiency, Chronic, Primary Health Care, Health Planning, Unified Health System, Epidemiology, Descriptive

## Abstract

**Objective:**

To determine the supply/demand ratio for procedures related to diagnosis and treatment for chronic kidney disease in the Brazilian National Health System (SUS), in the state of São Paulo, Brazil, 2019.

**Methods:**

This was a descriptive study, using data from the SUS outpatient and hospital information systems. The numbers of medical consultations, diagnostic and chronic kidney disease monitoring tests, performed in the period, were compared with the demand estimation, obtained through ministerial guidelines.

**Results:**

Exclusive SUS users were 28,791,244, and individuals with arterial hypertension and/or diabetes *mellitus*, 5,176,188. The number of procedures performed and the ratio between this number and the needs of the population were 389,414 consultations with nephrologists (85%); 11,540,371 serum creatinine tests (223%); 705,709 proteinuria tests (14%); 438,123 kidney ultrasounds (190%); and 1,045 kidney biopsies (36%).

**Conclusion:**

In the chronic kidney disease care in the SUS it could be seen simultaneous existence of lack of supply, waste and inadequate screening of important procedures.

**Study contributions**

**Main results**

In 2019, in public health care for chronic kidney disease in the state of São Paulo, it could be seen simultaneous existence of waste, lack of supply and poor screening of important procedures.

**Implications for services**

Periodic monitoring of supply/demand ratio for medical consultations and diagnostic tests by health services is essential to reduce the discrepancies found.

**Perspectives**

Better results can be obtained through computerized and integrated data systems, agreement between municipalities and health administrative regions, and professional training.

## Introduction

Chronic kidney disease is a worldwide public health problem. It affects about 10% of adults, 12% of people with arterial hypertension, 15% of those with diabetes *mellitus* and 30% of older adults. The World Health Organization (WHO) has defined this disease as the most neglected chronic non-communicable disease in the world. Globally, there is a lack of surveillance systems and national programs aimed at the treatment for early stages of chronic kidney disease. In addition, many countries have not included the disease in action plans for coping with chronic conditions and many people still die every year without access to dialysis.^1^

According to the International Society of Nephrology, chronic kidney disease is defined as kidney damage or glomerular filtration rate below 60 ml/min/1.73 m^2^ or by the presence of a marker of kidney damage for more than three months.^2^ Two simple, low-cost tests made available in the Brazilian National Health System (SUS) are sufficient for this diagnosis.^3^ One of these tests is serum creatinine. Estimated glomerular filtration rate is obtained from the serum creatinine test and using age, sex and ethnicity. This test measures the overall function of both kidneys. Another indispensable test used to diagnose chronic kidney disease is proteinuria (urinary protein level). This marker is the most important in clinical practice, because it changes earlier than the estimated glomerular filtration rate in the natural history of chronic kidney disease, and thus constitutes the main prognostic factor in individuals with this health condition. Compared to individuals with normal level proteinuria, those with alteration in this test have a higher risk of hospitalization, cardiovascular event and need for dialysis.^2^ ^,^ ^3^

The line of care for people with this health condition is well established by Brazilian and international guidelines.^2^ ^,^ ^3^ At the Primary Health Care (PHC) level, every individual with hypertension or diabetes should have a serum creatinine and proteinuria test performed at least annually. However, information on the adherence to these guidelines is scarce in Brazil. International studies have shown that less than 6% of individuals with chronic kidney disease are diagnosed in the early stages of the disease. These studies also showed that only 25% of cases underwent adequate screening in PHC and that late referral to the nephrologist was performed in 40% to 80% of them.^4^ ^,^ ^5^

The global agenda for coping with chronic kidney disease involves, in addition to professional training and individual awareness campaigns, the expansion of health care coverage.^1^ For this last action, determining the estimates of the needs of each population and monitoring the supply of health service is crucial.

In Brazil, the methodology for calculating the demand for health services in the SUS (medical consultations, tests, hospital beds, among others) is based on national and international scientific evidence, expert opinions and public consultations, culminating with Ordinance No. 1,631, of October 1, 2015, when the criteria and parameters of care for planning and promoting health actions and services within the SUS were approved.

Globally, regarding chronic kidney disease treatment, comparative studies of the number of medical consultations and diagnostic tests performed, with the needs of the population, are scarce. Therefore, the aim of this study was to determine the supply/demand ratio of procedures related to diagnosis and treatment for chronic kidney disease in the SUS, in the state of São Paulo, Brazil.

## Methods

### Study design

This was a descriptive study, based on data from the SUS Outpatient Information System (SIA/SUS) and the Hospital Information System (SIH/SUS). We analyzed the period from January 1^st^ December 31, 2019, in the state of São Paulo. The justification for choosing the period of 2019 was related to the fact that it was the most recent year without interference from the COVID-19 pandemic in the treatment of chronic diseases. Data were retrieved from both information systems between January and April 2021.

### Setting

SIA/SUS and SIH/SUS are secondary databases that store information about health care-related procedures (medical consultations, diagnostic tests, surgeries, high-cost medicines, hospitalizations, among others). These national systems cover all health facilities that provide services to the SUS and their objective is to collect information on the number of procedures performed. This information is sent electronically, on a monthly basis, from health facilities to the Ministry of Health, which is responsible for consolidating this information and publishing it on the Brazilian National Health System Information Technology Department (DATASUS) website, within 90 days. These are public domain data, which allow stratification by municipality, regional health department (administrative divisions of the SUS) or Federative Unit (FU).^7^

### Participants

**Figure 1:**
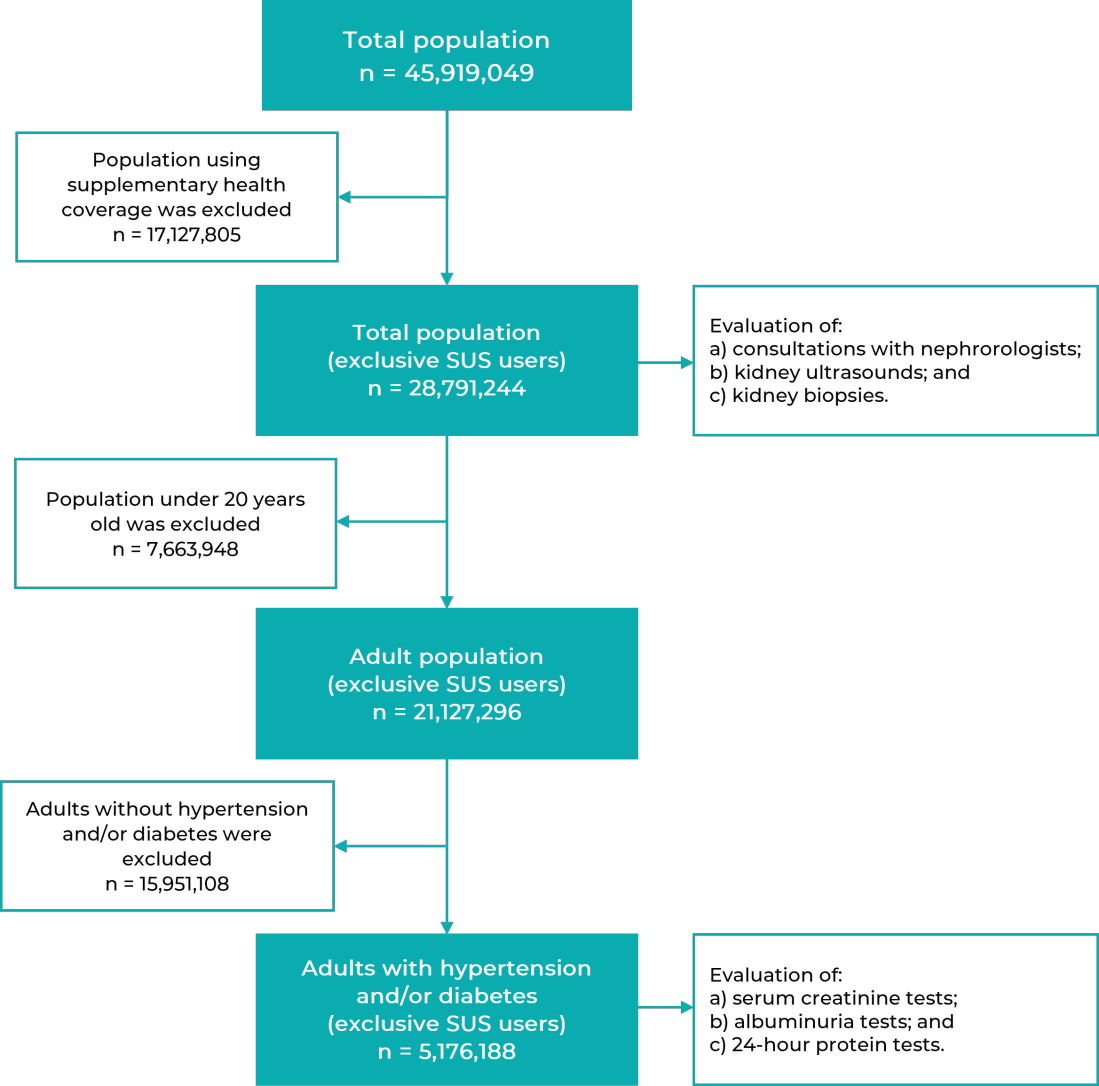
Process of inclusion of people assessed for consultations with nephrology specialists and chronic kidney disease examinations and diagnosis on the Brazilian National Health System, São Paulo State, 2019

The reference population of this study was the number of adults with hypertension and/or diabetes (supplementary health users were excluded), for the analysis of the supply/demand ratios of serum creatinine and proteinuria tests. For the analysis of supply/demand ratios of consultations with nephrologists, kidney ultrasounds and kidney biopsies, the total population of the state of São Paulo was considered as a reference population (population using the supplementary network was excluded) (Figure 1).

### Variables

Study variables were obtained for each regional health department and for the entire state of São Paulo. The choice of procedures related to diagnosis and treatment for chronic kidney disease was made according to the following criteria: scientific relevance; information availability in the SUS secondary databases; and the presence of demand estimation in current guidelines or ministerial ordinances. Sociodemographic, economic and structural characteristics of each regional health department were determined in order to relate them to the supply/demand ratio of diagnostic procedures and treatment for chronic kidney disease. The variables analyzed were:

- consultations with nephrologists (number of outpatient consultations with nephrologists in the SUS);
- serum creatinine tests (number of serum creatinine tests via the SUS);
- albuminuria tests (number of albuminuria tests via the SUS);
- 24-hour proteinuria tests (number of 24-hour proteinuria tests via the SUS);
- kidney ultrasounds (number of kidney ultrasound examinations performed via the SUS);
- kidney biopsies (number of kidney biopsies performed via the SUS);
- illiteracy rate (non-literate population over the total resident population);
- degree of urbanization (percentage of urban population over the total resident population);
- gross domestic product (GDP) *per capita* (in current BRL);
- average *per capita* income (in current BRL);
- density of nephrologists (number of nephrologists who have professional bond with the SUS, per 100,000 inhabitants);
- density of endocrinologists (number of endocrinologists who have professional bond with the SUS, per 100,000 inhabitants);
- density of general practitioners (number of general practitioners who have professional bond with the SUS, per 100,000 inhabitants);
- medical consultations in PHC (number of medical consultations in PHC performed by a medical professional in the year, per inhabitant);
- density of ultrasound machines (number of ultrasound machines used in the SUS, per 100,000 inhabitants).

### Data sources

The number of medical consultations and tests related to chronic kidney disease were retrieved from the SIA/SUS and SIH/SUS databases.^7^ The sociodemographic, economic and structural characteristics of the regional health departments were obtained from the Health Indicator Matrix of the São Paulo State Department of Health, and from the National Health Establishment Registry (CNES).^8^ ^,^ ^9^

### Measurement

An annual serum creatinine test and an annual proteinuria test for each adult with hypertension and/or diabetes were used as a demand estimation.^6^ The total number of adults in the state of São Paulo and in each regional health department was obtained from the Fundação Sistema Estadual de Dados (SEADE) website.^9^ The prevalence of adults with hypertension and diabetes was estimated at 23.0% and 8.0%, respectively.^10^ The combination of these two health conditions was estimated at 6.5%.^11^ Therefore, for the calculation of the number of people with hypertension and/or diabetes, the study considered a prevalence of 24.5% of adults. Thus:

Demand for creatinine tests = 1 x No. of adults x 24.5%

Demand for proteinuria tests = 1 x No. of adults x 24.5%

Demand for consultations with nephrologists was estimated at 1,600 consultations/100,000 inhabitants of the total population, regardless of age or comorbidities, and demand for kidney ultrasounds, estimated at 800/100,000 inhabitants.^6^ The estimated demand for kidney biopsies for this study was empirically established at 10 biopsies per 100,000, given that it is the average of values observed in the literature.^11^ Therefore:

Demand for consultations with nephrologists = 1,600 x No. of inhabitants/100,000

Demand for kidney ultrasounds = 800 x No. of inhabitants/100,000

Demand for kidney biopsies = 10x No. of inhabitants/100,000

Supply/demand ratio for medical consultations and procedures related to chronic kidney disease was expressed in percentages and calculated according to the following steps:

Supply = No. of procedures performed

Demand = No. of procedures required for each population

Supply/demand ratio (%) = (supply/demand) x 100

### Bias control

In order to calculate the demand for each procedure analyzed in this study, the total population and the population with hypertension and/or diabetes were reduced from the percentage of supplementary health coverage in each regional health department and in the state of São Paulo. This information was obtained from Agência Nacional de Saúde Suplementar (ANS) website, the national regulatory agency for private health insurance and plans in Brazil.^12^ The formula used to calculate the reference population was:

Reference population = population x (1 - % supplementary health coverage)

### Statistical methods

The study variables were recorded in modeling spreadsheets using the Microsoft Excel software, version 2019. The number of adults with hypertension and/or diabetes, the number of procedures required (according to pre-established parameters) and supply/demand ratios were also calculated using Microsoft Excel software.

The correlations between supply/demand ratios for each procedure related to chronic kidney disease, and the sociodemographic, economic and structural variables of the regional health departments, were calculated by means of Spearman's rank correlation test (Software SPSS Inc., Chicago, IL, USA, version 19.0). The significance level adopted for this coefficient was 5%.

### Ethical aspects

The study project was exempted from submission to a Research Ethics Committee (REC), given that it used secondary data, in the public domain, without personal identification.

## Results

The most populous regional health departments with the highest number of adults with hypertension and/or diabetes in the state of São Paulo, in 2019, were those in the metropolitan area of São Paulo (21,734,682 inhabitants and 3,865,442 adults with hypertension and/or diabetes) and Campinas (4,671,287 and 847,930, respectively). The least populated departments with the lowest number of adults with hypertension and/or diabetes were Registro (284,509 and 49,539, respectively) and Barretos (440,907 and 80,893, respectively). The departments with the highest supplementary health coverage, in 2019, were those in the metropolitan area of São Paulo (43.0%) and Campinas (41.8%), and those with the lowest coverage were in Registro (9.0%) and Marília (19.8%) (Table 1).

**Table 1:** Total population, 20 years and older, adults with hypertension and/or diabetes *mellitus* and using supplementary health coverage, by regional health department, state of São Paulo, 2019

Regional health department	Total population^a^	20 years and older population^a^	Adults with hypertension and/or diabetes^a,b^	Percentage of supplementary health coverage^c^
Metropolitan area of São Paulo	21,734,682	15,777,313	3,865,442	43.0
Araçatuba	791,256	602,709	147,664	23.5
Araraquara	1,025,982	767,356	188,002	35.5
Baixada Santista	1,865,397	1,359,370	333,046	36.8
Barretos	440,907	330,176	80,893	29.5
Bauru	1,800,757	1,332,249	326,401	23.3
Campinas	4,671,287	3,460,938	847,930	41.8
Franca	718,176	521,324	127,724	31.7
Marília	1,149,132	866,792	212,364	19.8
Piracicaba	1,586,546	1,174,604	287,778	40.9
Presidente Prudente	775,627	586,950	143,803	22.8
Registro	284,509	202,198	49,539	9.0
Ribeirão Preto	1,523,682	1,124,229	275,436	36.9
São João da Boa Vista	834,872	630,473	154,466	29.7
São José do Rio Preto	1,629,470	1,248,033	305,768	30.4
Sorocaba	2,534,157	1,844,086	451,801	27.7
Taubaté	2,552,610	1,867,047	457,427	29.4
**State of São Paulo**	**45,919,049**	**33,695,847**	**8,255,483**	**37.3**

a) Fundação Sistema Estadual de Dados (SEADE); b) Chronic Disease Risk and Protective Factors Surveillance Telephone Survey (VIGITEL Brasil 2019); c) Agência Nacional de Saúde Suplementar (ANS).

In 2019, 389,414 consultations with nephrologists were performed via the SUS, throughout the state of São Paulo, where there was a supply of 11,540,371 serum creatinine tests, and 412,772 albuminuria tests, 292,937 24-hour proteinuria tests, 438,123 kidney ultrasounds and 1,045 kidney biopsies were performed. The supply/demand ratio for consultations with nephrologist in the state was 84.6%. The regions where it was higher were Taubaté (205.9%) and Barretos (146.2%), and it was lower in Piracicaba (36.8%) and Araraquara (44.6%). The supply/demand ratio for serum creatinine tests was 223.0% in the state of São Paulo, and the regions with the highest rates were Barretos (348.4%) and the metropolitan area of São Paulo (262.7%), and the regions with the lowest rates were Araçatuba (70.1%), Araraquara (146.6%) and Presidente Prudente (147.4%) (Table 2).

**Table 2:** Absolute number and supply/demand ratio for consultations with nephrologists, laboratory tests and procedures related to chronic kidney disease, by regional health department, for the population using the Brazilian National Health System (n = 28,791,244), state of São Paulo, 2019

Regional health departament	n (%)^a^					
	Consultation with nephrologists	Serum creatinine	Albuminuria	24-hour proteinuria	Kidney ultrasounds	Kidney biopsies
Metropolitan area of São Paulo	129,717 (65.4)	5,793,701 (262.7)	199,533 (9.0)	159,150 (7.2)	215,650 (217.4)	733 (59.1)
Araçatuba	4,726 (48.8)	79,183 (70.1)	2,386 (2.1)	1,663 (1.5)	6,014 (124.1)	1 (1.7)
Araraquara	4,725 (44.6)	177,792 (146.6)	4,012 (3.3)	3,814 (3.1)	8,870 (167.6)	0 (0.0)
Baixada Santista	20,764 (110.0)	432,517 (205.4)	16,292 (7.7)	7,860 (3.7)	18,530 (196.4)	3 (2.5)
Barretos	7,273 (146.2)	198,692 (348.4)	4,980 (8.7)	3,591 (6.3)	5,162 (207.6)	2 (6.4)
Bauru	20,851 (94.3)	429,834 (171.6)	10,831 (4.3)	21,188 (8.5)	12,976 (117.4)	26 (18.8)
Campinas	36,101 (83.0)	1,036,812 (210.1)	63,506 (12.9)	29,257 (5.9)	39,441 (181.3)	79 (29.1)
Franca	7,840 (99.8)	137,917 (158.0)	6,189 (7.1)	1,582 (1.8)	9,471 (241.2)	8 (16.3)
Marília	8,810 (59,7)	264,126 (155.0)	3,883 (2.3)	3,402 (2.0)	8,577 (116.3)	20 (21.7)
Piracicaba	5.522 (36.8)	360,660 (212.1)	7,824 (4.6)	4,940 (2.9)	9,896 (132.0)	4 (43)
Presidente Prudente	5,703 (59.5)	163,686 (147.4)	4,687 (4.2)	2,109 (1.9)	9,060 (189.1)	26 (43.4)
Registro	2,025 (48.9)	99,276 (220.2)	997 (2.2)	461 (1.0)	5,306 (256.1)	3 (11.6)
Ribeirão Preto	21,366 (138.9)	443,680 (255.2)	20,457 (11.8)	10,520 (6.1)	11,770 (153.0)	81 (84.2)
São João da Boa Vista	5,551 (59.1)	171,300 (157.8)	3,676 (3.4)	2,686 (2.5)	7,993 (170.3)	4 (6.8)
São José do Rio Preto	25,191 (138.9)	408,133 (191.8)	11,385 (5.4)	13,184 (6.2)	24,286 (267.8)	13 (11.5)
Sorocaba	23,883 (81.4)	577,184 (176.6)	19,633 (6.0)	10,124 (3.1)	20,402 (139.2)	12 (6.5)
Taubaté	59,366 (205.9)	765,878 (237.2)	32,501 (10.1)	17,406 (5.4)	24,719 (171.5)	30 (16.7)
**State of São Paulo**	**389,414 (84.6)**	**11,540,371 (223.0)**	**412,772 (8.0)**	**292,937 (5.7)**	**438,123 (190.3)**	**1,045 (36.3)**

a) We presented absolute numbers, and the supply/demand ratio in percentage, in parenthesis.

For the whole state of São Paulo, albuminuria tests represented 8.0% of the estimated demand, and 24-hour proteinuria tests, 5.7%. The regional health departments with the highest supply/demand ratios for albuminuria were Campinas (12.9%) and Ribeirão Preto (11.8%), and those where these relationships were lower in Araçatuba (2.1%), Registro (2.2%) and Marília (2.3%) and Araraquara (3.3%). The highest supply/demand ratios for 24-hour proteinuria were found for Bauru (8.5%) and the metropolitan area of São Paulo (7.2%), and the lowest for Registro (1.0%) and Araçatuba (1.5%). Throughout the state, the number of ultrasounds performed represented 190.3% of the estimated demand for São Paulo’s population, with the highest percentages in the regions of São José do Rio Preto (267.8%) and Registro (256.1%), and the lowest in the regions of Marília (116.3%) and Bauru (117.4%). The number of kidney biopsies performed in the state represented 36.3% of the estimated demand, and the regional health departments with the highest supply/demand ratios for this procedure were those in Ribeirão Preto (84.2%) and the metropolitan area of São Paulo (59.1%), and the departments with the lowest rates were those in Araraquara (0.0%) and Araçatuba (1.7%) (Table 2).

The highest illiteracy rates were observed in Registro (8.5%) and Presidente Prudente (6.8%), and the lowest in the metropolitan area of São Paulo (3.5%) and Taubaté (3.8%). The highest degree of urbanization was found for Baixada Santista (99.8%) and the metropolitan area of São Paulo (98.9%), and the lowest for Registro (71.2%) and Sorocaba (86.3%). The highest GDP per capita was found for Campinas (BRL 65,048.00) and the metropolitan area of São Paulo (BRL 55,053.00), and the lowest was found for Presidente Prudente (BRL 29,387.00) and Registro (BRL 30,831.00) (Table 3).

**Table 3:** Sociodemographic, economic and structural characteristics of regional health departments, state of São Paulo, 2019

Regional health departament	Illiteracy rate (%)	Degree of urbanization (%)	GDP^a^ *per capita* (BRL)	*Per capita* income (BRL)	Density of nephrologists^b^	Density of endocrinologists^b^	Density of general practitioner^b^	Medical consultations in PHC^c^/year/inhabitant	Density of ultrasound machinesd
Metropolitan area of São Paulo	3.5	98.9	55,053	1,175.0	2.7	1.8	52.9	1.1	7.3
Araçatuba	5.9	92.3	31,007	817.7	0.6	1.0	78.5	1.9	9.4
Araraquara	5.0	95.3	38,041	888.9	1.7	3.1	116.4	1.0	7.9
Baixada Santista	4.0	99.8	34,319	967.8	2.4	1.4	79.4	0.7	7.9
Barretos	5.6	94.8	41,710	803.9	3.2	3.2	127.0	2.3	11.6
Bauru	5.3	91.9	32,840	857.4	2.7	2.1	85.9	1.2	9.8
Campinas	3.9	95.6	65,048	1,073.4	2.2	1.6	70.8	0.8	6.5
Franca	5.0	95.2	33,115	816.1	1.7	1.7	85.5	1.5	9.7
Marília	6.0	91.4	31,020	791.1	1.3	3.0	107.3	1.5	9.2
Piracicaba	4.2	95.3	45,468	938.5	2.2	1.0	110.4	1.0	7.3
Presidente Prudente	6.8	89.3	29,387	795.2	1.8	1.3	121.7	1.5	8.5
Registro	8.5	71.2	30,831	524.2	0.7	0.4	52.0	1.1	15.1
Ribeirão Preto	4.6	97.0	41,833	1,014.7	3.2	2.0	84.6	1.6	12.4
São João da Boa Vista	5.2	90.7	34,215	819.5	1.6	1.8	76.8	1.5	7.8
São José do Rio Preto	5.8	91.7	33,611	887.8	2.0	2.1	108.3	2.9	10.5
Sorocaba	4.9	86.3	38,333	798.5	1.9	2.0	58.1	1.1	6.9
Taubaté	3.8	94.1	45,475	910.4	2.4	1.9	86.5	1.0	8.5
**State of São Paulo**	**4.2**	**95.9**	**48,538**	**1,036.5**	**2.4**	**1.8**	**70.3**	**1.2**	**7.9**

a) GDP: Gross domestic product; b) Number of professionals linked to the Brazilian National Health System (SUS), per 100,000 inhabitants; c) PHC: Primary Health Care; d) Number of devices in use in the SUS, per 100,000 inhabitants.

The locations with the highest densities of nephrologists were Barretos (3.2) and Ribeirão Preto (3.2), and those with the lowest densities of these professionals, were Araçatuba (0.6) and Registro (0.7). It could be seen the highest densities of general practitioners in Barretos (127.0) and Presidente Prudente (121.7), and the lowest in Registro (52.0) and the metropolitan area of São Paulo (52.9). The health regions with the highest average of medical consultations in PHC/inhabitant/year were São José do Rio Preto (2.9) and Barretos (2.3), and those with the lowest average were Baixada Santista (0.7) and Campinas (0.8). The density of ultrasound machines was higher in Registro (15.1) and Ribeirão Preto (12.4), and lower in Campinas (6.5) and Sorocaba (6.9) (Table 3).

Supply/demand ratio for consultations with nephrologists showed a direct correlation with the density of nephrologists (r = 0.64; p-value = 0.004). Supply/demand ratio for serum creatinine tests showed an inverse and significant correlation with illiteracy rate (r = -0.51; p-value = 0.031), and a direct correlation with GDP per capita (r = 0.67; p-value = 0.002) and density of nephrologists (r = 0.75; p-value < 0.001). Supply/demand ratio for proteinuria tests showed an inverse and significant correlation with illiteracy rate (r = -0.71; p-value = 0.001), and direct correlation with the total population (r = 0.64; p-value = 0.004), degree of urbanization (r = 0.63; p-value = 0.005), GDP per capita (r = 0.79; p-value < 0.001), per capita income (r = 0.72; p-value = 0.001) and density of nephrologists (r = 0.85; p-value < 0.001) (Table 4).

**Table 4:** Correlations between the supply and demand for consultations with nephrologists, tests that are relevant to chronic kidney disease and sociodemographic, economic and structural variables for the population using the Brazilian National Health System (n = 28,791,244), state of São Paulo, 2019

Supply/demand ratio	r^a^ (p-valor^b^)^c^									
	Population	Illiteracy rate	Degree of urbanization	GDP^a^ *per capita*	*Per capita* income	Density of nephrologists^b^	Density of endocrinologists^b^	Density of general practitioner^b^	Medical consultations in PHC^c^/year/inhabitant	Density of ultrasound machinesd
Consultations with nephrologists	0.26 (0.287)	-0.28 (0.266)	0.25 (0.324)	0.28 (0.257)	0.21 (0.404)	0.64 (0.004)	0.43 (0.077)	0.13 (0.610)	0.22 (0.389)	0.32 (0.200)
Serum creatinine	0.34 (0.163)	-0.51 (0.031)	0.44 (0.066)	0.67 (0.002)	0.45 (0.060)	0.75 (< 0.001)	0.10 (0.781)	-0.20 (0.414)	-0.18 (0.483)	0.08 (0.754)
Proteinuria	0.64 (0.004)	-0.71 (0.001)	0.63 (0.005)	0.79 (< 0.001)	0.72 (0.001)	0.85 (< 0.001)	0.32 (0.192)	-0.06 (0.817)	-0.17 (0.509)	-0.16 (0.531)
Kidney ultrasounds	-0.09 (0.705)	-0.04 (0.864)	0.18 (0.645)	0.07 (0.773)	0.08 (0.760)	0.15 (0.559)	-0.17 (0.508)	-0.15 (0.559)	0.04 (0.867)	0.20 (0.433)
Kidney biopsies	0.32 (0.191)	-0.16 (0.520)	0.12 (0.622)	0.20 (0.418)	0.24 (0.336)	0.33 (0.179)	0.02 (0.938)	-0.22 (0.372)	0.10 (0.680)	0.08 (0.742)

a) r: Correlation coefficient; b) Spearman’s rank correlation test; c) we presented correlation coefficients, and the p-value of the correlations, in parenthesis; d) GDP: Gross domestic product; e) PHC: Primary Health Care.

## Discussion

The study points to a simultaneous existence of waste and lack of different procedures related to the diagnosis and monitoring of chronic kidney disease via the SUS in the state of São Paulo. If, on the one hand, the number of serum creatinine tests offered and kidney ultrasounds performed was higher than the estimated needs of the population, on the other hand, urinary protein tests, kidney biopsies and consultations with nephrologists were lower than the number considered adequate. In addition, it was possible to identify important differences between the regional health departments in the state with regard to these parameters.

Some limitations of this study should be taken into consideration. Initially, the analysis was performed based solely on the number of medical consultations and diagnostic tests reported by health care providers in the SUS, in the state of São Paulo, and it was not possible to evaluate absenteeism or waiting list. In addition, the available databases did not allow identifying the characteristics of the requesting professionals (PHC physicians or specialists), the demographic data and the comorbidity profile of the individuals who had medical consultations and underwent diagnostic tests. Finally, the secondary data source condition of this study did not allow the evaluation of duplicate medical consultations or diagnostic tests. Moreover, it is possible that supply/demand ratios for medical consultations and diagnostic tests indicated for individuals with kidney disease are not the only factors related to important outcomes that have not been measured in this study, such as hospitalizations, case fatality ratio and planned initiation of dialysis. It is also worth mentioning that quality of care indicators (such as percentage of blood pressure and diabetes control, and use of renin-angiotensin system blockers) and the provision of a multidisciplinary team (nutritionist, psychologist, nurse, social worker) are known as influencers of outcomes in people with chronic kidney disease.^1^ ^,^ ^15^ ^,^ ^16^

The number of serum creatinine tests and kidney ultrasounds performed in the state was about twice as high as the estimated demand. This finding may be related to the repetition of diagnostic tests. Moreover, it is possible that the lack of electronic medical records and fragmentation of health care via the SUS make it difficult the rationalization of use of supplementary diagnostic tests.^17^ Corroborating the results presented here, few studies previously conducted in the country showed the unnecessary use of supplementary diagnostic tests in specific scenarios such as: the care of people with hypertension and diabetes in PHC, the routine preoperative for cataract surgery and the follow-up of individuals with low back pain.^18^ ^-^ ^20^ Similarly, previous studies indicated a significant increase in the number of high complexity imaging tests in Brazil and worldwide.^21^ ^,^ ^22^ A greater access to health care and technology, especially in regions with the highest rates of urbanization, associated with the production payment model, may explain this increase.

The number of creatinine tests obtained from Primary Health Care Information System (SIA/SUS) includes diagnostic tests performed on people undergoing renal replacement therapy. This fact could be another reason for the excess of diagnostic tests observed. However, taking into consideration that the estimated number of people on dialysis via the SUS in the state of São Paulo is 19,000, and that these system users undergo creatinine dosage on a monthly basis, the percentage would reach only 2.0% of the number performed (228,000 dosages out of 11,140,371).^3^

Unlike what was observed in the evaluation of the number of serum creatinine tests performed, renal function evaluation by means of proteinuria tests was less than 20% of the estimated demand (when considering the sum of the two most specific methods available in the SUS). Probably, this finding was due to the low request rate for proteinuria, given that it is a simple, available and low-cost test.^7^ In a representative sample of individuals receiving care via Medicare, the public health system in the United States, while the probability of a person with hypertension or diabetes having an annual serum creatinine test was nearly 100%, the probability of albuminuria testing represented only 30%.^23^ The low proteinuria test rates observed in this and other places probably reflect the low knowledge of general practitioners and non-nephrologists about (i) the importance of this test as a prognostic factor and (ii) the current definition and classification of chronic kidney disease.^24^ It is noteworthy that the Brazilian public policy aimed at addressing the most prevalent chronic diseases, such as hypertension and diabetes *mellitus*, determines that serum proteinuria and creatinine tests should be requested by non-nephrologists in PHC, with the purpose of screening for kidney injury.^6^ ^,^ ^25^

The regional differences between supply/demand ratios for procedures related to kidney disease, pointed out in this study, can be explained, although in part, by the sociodemographic and economic characteristics of the health departments. In fact, supply/demand ratios for creatinine and proteinuria tests found, showed an inverse correlation with illiteracy rates and direct association with GDP per capita, corroborating previous studies.^23^ ^,^ ^26^ Higher level of education and better financial and social conditions may be related to greater knowledge about chronic diseases, easier access to health units and, consequently, greater use of laboratory tests. In addition, the density of nephrologists was significantly correlated with the supply/demand ratio for creatinine and proteinuria tests, indicating the possibility of these tests being requested, proportionally, more frequently in specialized care and less frequently in PHC. Other possible explanations, although they have not been evaluated in this study, would be the differences in the prevalence of hypertension and diabetes, in addition to care practices, between the health regions in the state of São Paulo.

The small number of consultations with nephrologist and kidney biopsies performed in the state of São Paulo can be attributed to the small number of professionals in this specialty, as well as to late referral, given that chronic kidney disease is oligosymptomatic in its early stages and there are mistakes in its identification in PHC.^27^ ^,^ ^28^ Between 2008 and 2018, while the number of nephrologists in Brazil increased by 25%, the estimated number of people undergoing dialysis increased by 52%.^27^ This trend seems to be a worldwide phenomenon; in the United States, for example, between 1996 and 2012, the number of nephrologists per 1,000 individuals undergoing dialysis dropped from 18 to 10.^28^

Late referral to the nephrologist is probably also associated with a smaller number of kidney biopsies performed, a procedure that is not frequently indicated for cases of advanced stages of kidney disease.^29^ According to the 2018 Brazilian Dialysis Census, the percentage of people undergoing dialysis whose kidney disease etiology was identified as glomerulopathy, was at least 10%; however, the same Census found another 10% of individuals with chronic kidney disease 'of unknown etiology', a proportion that may encompass more cases of glomerulopathies, among other diseases that have not been diagnosed early due to late referral.^30^

Taking these results, it can be concluded that there is simultaneous waste and lack of medical consultations and diagnostic tests related to chronic kidney disease treatment in the state of São Paulo. This analysis can be an auxiliary tool for planning and decision making. It is necessary to evaluate measures in order to correct the discrepancies found, aiming to improve the efficiency of care of people with chronic kidney disease and those at higher risk of contracting the disease.
